# The Role of Dentists in COVID-19 Is Beyond Dentistry: Voluntary Medical Engagements and Future Preparedness

**DOI:** 10.3389/fmed.2020.00566

**Published:** 2020-10-06

**Authors:** Chaminda Jayampath Seneviratne, Matthew Wen Jian Lau, Bee Tin Goh

**Affiliations:** ^1^National Dental Research Institute Singapore, National Dental Centre Singapore, Singapore, Singapore; ^2^Duke-NUS Graduate Medical School, Singapore, Singapore

**Keywords:** COVID-19, dentistry, voluntary work, preparedness, infection control

## Abstract

The emergence of the highly infectious novel coronavirus SARS-CoV-2 has led to a global COVID-19 pandemic. Since the outbreak of COVID-19, worldwide healthcare systems have been severely challenged. The rapid and explosive surge of positive cases has significantly increased the demand for medical care. Herein we provide a perspective on the role dentists can play in voluntary medical assistance and future preparedness for a similar pandemic. Though dentists and physicians have different scopes of practice, their trainings share many similarities. Hence, dental professionals, with their knowledge of basic human science and sterile surgical techniques, are an invaluable resource in the COVID-19 pandemic response. Overall, it is commendable that many dentists have risen to the challenge in the fight against COVID-19. For example, in Singapore, National Dental Centre Singapore (NDCS) deployed dental clinicians as well as volunteers from research laboratories to screen for suspected cases, provide consultations as well as conduct swabbing operations. Dental practice will be considerably changed in the post-COVID-19 era. There is a greater need to have refresher courses for practicing dentists on new infection control strategies. Moreover, the curriculum in dental schools should be expanded to include competencies in pandemic and disaster relief. In addition, voluntary medical work should be made a part of the community dentistry curriculum. This volunteerism will leave a positive impact on developing the careers of young dentists. Hence, the contribution of dentists beyond dental practice in this pandemic situation will be appreciated by future generations.

## Background

The emergence of the highly infectious novel coronavirus has led to a global pandemic in a span of just 3 months. It was only on 31 December 2019 that first reports of pneumonia of an unknown cause detected in Wuhan, China, began to surface to the World Health Organization (WHO). Provisionally named as 2019 novel coronavirus (2019-nCoV), there was evidence of exponential human-to-human transmission in the early outbreak stage (1–3). Consequently, on 30 January 2020, the WHO declared the outbreak a Public Health Emergency of International Concern (PHEIC). Subsequently, assessment by the Coronavirus Study Group (CSG) of the International Committee on Taxonomy of Viruses found that SARS-CoV-2 clusters phytogenetically with the species *Severe acute respiratory syndrome-related coronavirus* (SARS-CoVs) and genus *Betacoronavirus*, and formally designated it as *Severe acute respiratory syndrome coronavirus 2* (SARS-CoV-2) (4). CSG also emphasized that the name SARS-CoV-2 has no relationship to the name of the SARS disease caused by SARS CoV-1. In fact, the SARS CoV-2 genome shares only 79.6% similarity to that of SARS-CoV-1 (5). The initial sequencing of the new coronavirus revealed that it is closely related to betacoronaviruses of bat origin i.e., bat-SL-CoVZC45 (87.99% similarity) and bat-SL-CoVZXC21 (87.23% similarity) (5). More recent genome sequencing showed that a bat coronavirus BatCoV RaTG13 originally found in the bat *Rhinolophus affinis* from Yunnan Province, China has 96.2% genome similarly to that of SARS CoV-2 (6). These foregoing studies indicate that the bats are the likely zoonotic reservoir host for SARS CoV-2. Although bats may be the original source of the new coronavirus, it is assumed to be transmitted to humans via an intermediate host, possibly pangolins or wild animals currently yet unknown (7). On 11 February 2020, the WHO announced coronavirus disease 2019 (COVID-19) to be the disease caused by SARS-CoV-2. Subsequently, many countries continued to experience clusters of cases and community transmissions. This led the WHO to declare the COVID-19 outbreak a pandemic on 12 March 2020.

## Global Havoc Caused by COVID-19

As of 26 July 2020, more than 15.78 million confirmed cases of COVID-19 and 640,016 associated deaths have been reported worldwide (8). With the number of infected cases surging each day, researchers are racing to understand what makes it spread so easily. From the evidence so far, the transmission of SARS-CoV-2 can occur via respiratory droplets, contact and aerosols (9). A major factor facilitating the transmission of COVID-19 is the high level of SARS-CoV-2 shedding in the upper respiratory tract, even among pre-symptomatic patients (10). It has been reported that pharyngeal virus shedding of SARS-CoV-2 reaches its peak on day 4 of symptoms onset, and it can be over 1,000 times higher than SARS-CoV-1 (11). Numerous cases have been reported wherein patients who had positive test results were asymptomatic at testing (12). Moreover, the asymptomatic incubation period for infected individuals could be ~1–14 days in general, although longer incubation periods as long as 24 days have been reported. Thus, symptom-based screening alone may have failed to detect a high number of COVID-19 positive cases and most likely contributed to the rampant transmission.

## Impact of COVID-19 on Healthcare Providers

Since the outbreak of COVID-19, worldwide healthcare systems have been severely challenged. The rapid and explosive surge of positive cases have led to a significant increase in the demand for medical care. On the infrastructure front, hospitals have actively scaled up their capacity of basic and critical care beds. However, global medical manpower resources are finite. Consequently, many hospital-based healthcare workers have had to work over-hours and take on extra shifts. Such stressors have been associated with reduced job performance and fatigue-related errors which could harm patients (13). In responding to this crisis with a multi-sectorial, equitable and human-rights focused approach, the United Nations entities have called for voluntary support from professionals with medical backgrounds for various job capacities to manage the pandemic (14).

## Are Dentists Equipped to Support Workforce Working Against COVID-19?

Though dentists and physicians have different scopes of practice, their trainings share many similarities. The dental student, like his medical counterpart, has to attain proficiency in his understanding of the basic sciences such as anatomy, physiology, pharmacology, and microbiology. This is essential given that dentists are expected to competently manage dental issues of medically compromised patients. Moreover, dentists must be able to expeditiously and effectively manage medical emergencies that may arise in routine dental practice. To this end, many dental practitioners would have undergone basic cardiac life support training. Thus, the robust training of clinical medicine in dentistry strengthens the candidature of dentists to volunteer services for COVID-19 control and spread.

The outbreak of COVID-19 has significantly affected the practice of dentistry. Dental treatment can generate large amounts of aerosols and droplets mixed with the patient's saliva or blood (15). This poses a risk to dental professionals as SARS-CoV-2 has been detected in saliva of infected individuals (16). Many dentists have therefore discontinued the provision of elective dental treatment, in accordance with guidelines released by national-level government healthcare authorities such as the Centers for Disease Control and Prevention (CDC) in the US and National Health Service (NHS) in the UK. Only limited cases that require urgent or emergency dental care continue to be seen. The significantly reduced workload during this time, coupled with robust training in a medical setting, makes the dentist a prime candidate to volunteer in the fight against COVID-19.

## How Can the Dental Fraternity Play Their Part in the Current Crisis?

Dental professionals, with their knowledge of basic human science and sterile surgical techniques, are an invaluable resource in the COVID-19 pandemic response. Licensed dentists are eligible to administer COVID-19 diagnostic tests such as nasopharyngeal and oropharyngeal swabs. With their detailed understanding of head and neck anatomy, dentists are well placed to perform such procedures accurately and atraumatically. This is imperative as irritation to the oral or nasal mucosa while swabbing risks the patient sneezing or coughing, potentially releasing contaminated droplets and aerosols to the environment. Unlike negative-pressure rooms, many makeshift medical screening facilities are unable to limit the aerosol spread of SARS-CoV-2. This may lead to contamination of open-air healthcare facilities. In this context, dental clinics that are well equipped with facilities to control aerosol spread of infections, such as negative pressure rooms and high-volume excavators, can offer help to augment the capacity for COVID-19 screening. In this regard, global health authorities as well as health ministries from the respective countries have provided clear standard infection control procedures for dentists (17–19).

Dentists can also assist their medical counterparts in the inpatient setting. Such duties include patient triage, monitoring vital signs, administering oxygen and injectables, and writing prescriptions. Should emergency procedures need to be performed, dentists are capable of administering local anesthesia and suturing. In addition, oral surgeons and dentist anesthesiologists are competent in performing intubation, deep sedation and general anesthesia services (20).

The COVID-19 outbreak as well as harsh lock down practices worldwide have created a stressful environment for many people globally. Such stressful situations have been shown to lead to poor oral health (21, 22). Therefore, oral healthcare professionals should consider developing online platforms to provide information on oral hygiene and oral health maintenance. Digitalized healthcare services can be implemented with a qualified team of dentists being available online to provide reliable oral healthcare solutions in an accessible, affordable and appropriate manner and allay patients' dental concerns during the lockdown period. Oral healthcare professionals can also engage in voluntary service for residents in community housing to promote good oral health. The recommendations on how the dental fraternity can play their part in the current crisis are summarized in Table 1.

**Table 1:** Key considerations and recommendations on how dental fraternity can play their part in the current crisis and recommendations for dental implementation in post-COVID-19 era.

**Key considerations**	**Recommendations**
How dental fraternity can play their part in the current crisis	Administer COVID-19 diagnostic tests • Nasopharyngeal swabs • Oropharyngeal swabs • Salivary sampling
	Volunteer appropriately equipped dental clinics as screening facility • Negative pressure rooms • High-volume excavators
	Assist medical counterparts in the inpatient setting
	Develop online platforms to promote oral hygiene and oral health maintenance
Recommendations for dental implementation in post-COVID-19 era	Revise and strengthen education on infection control measures in dental practice
	Dental education should include competencies in pandemic and disaster relief • Training to administer COVID-19 diagnostic tests • Training to be part of an effective surveillance network
	Include voluntary medical work in the community dentistry curriculum
	Organize refresher courses for practicing dentists on infection control and best practices

## Evidence Reports of Volunteering Acts by Dentists on the Global Forefront

**Figure 1:**
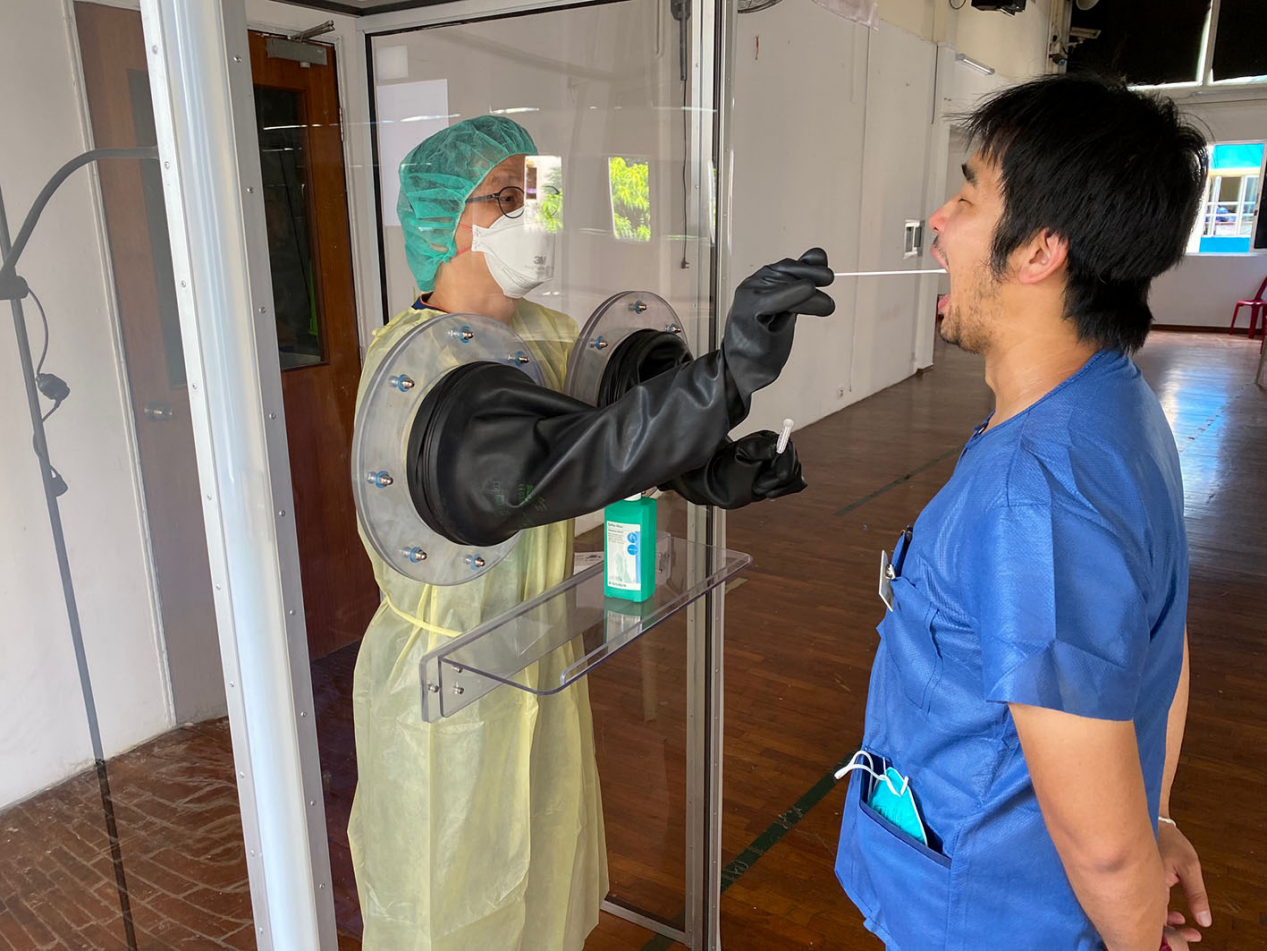
Dental clinicians from National Dental Centre Singapore were trained to conduct swab procedure using swab booths manufactured in Singapore (Photo credit: National Dental Centre Singapore).

**Figure 2:**
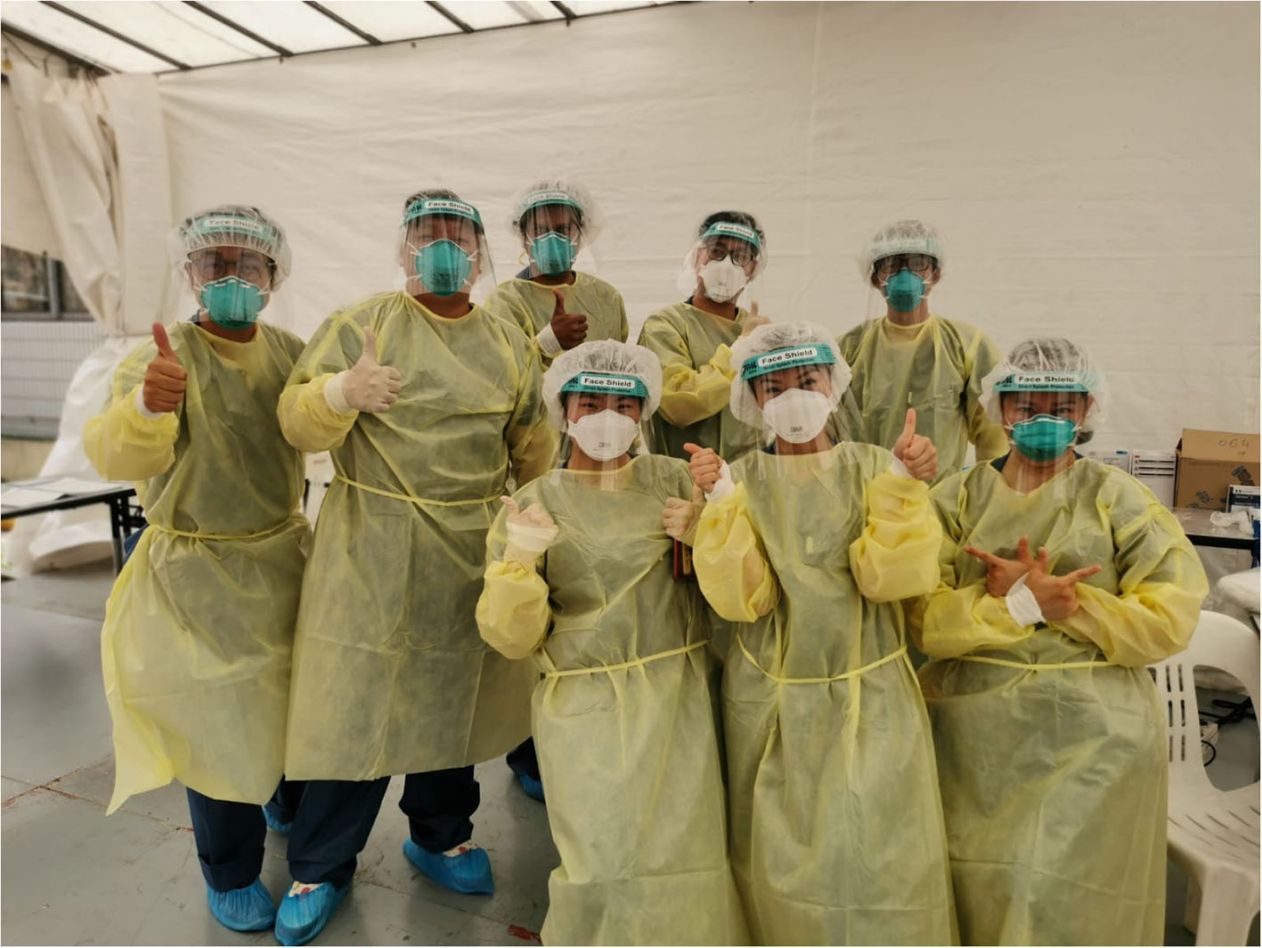
A team of Singapore Health Services (SingHealth) volunteers assigned to manage a medical clinic at a foreign workers' dormitory in Singapore. This team included a neurologist, a dental scientist, a pharmacologist, a cancer patient service associate, a radiographer and nurses (Photo credit: National Dental Centre Singapore).

In Singapore, while limited community transmission of COVID-19 remains, there has been a rapid surge in the number of infected cased among foreign workers living in dormitories. Such facilities become conducive for rapid spread of SARS-CoV-2 as residents are housed in close proximity and share many common amenities. In a whole-of-government approach to isolate and eradicate the virus, the Singapore government implemented aggressive mass-scale testing for COVID-19 for foreign workers residing in dormitories. This major operation was undertaken by multiple agencies including Singapore Health Services (SingHealth), Singapore Police Force and Singapore Armed Forces. Supporting this move, National Dental Centre Singapore (NDCS) deployed dental clinicians as well as volunteers from research laboratories to the foreign worker dormitories to conduct swab operations. All patients were treated as suspect cases, and all volunteers observed universal precautions and donned appropriate personal protective equipment (PPE). The use of specially manufactured swab booths further minimized the risk of cross-infection between patients and volunteers (Figure 1). In this massive operation, NDCS staff worked collaboratively with colleagues across various professional backgrounds including clinicians, nurses, pharmacists, radiographers, and medical social workers (Figure 2). Without the cohesive and coordinated effort, it would have been a considerable task to successfully establish and staff the field swab clinics within a short period of time.

In the UK, dentists, dental support teams as well as clinical academics have played a vital role in supporting the NHS (23). For instance, there are news reports that dental staff from Bath Health NHS Trust and QMUL Institute of Dentistry are helping in maternity, critical care, and emergency units. Dental hospitals have undergone reconfiguration to support medical care. In the US, dentist volunteers in states such as Virginia and California have responded to appeals to assist with critical emergency care needs, and have been redeployed to the frontlines. Importantly, states such as California have made changes to their law to allow greater flexibility in scope and licensure in the time of a catastrophic emergency. The law provides immunity from liability for care provided “in good faith” during an emergency for a person who “voluntarily and without compensation or expectations of compensation, and consistent with the dental education and emergency training that he or she has received, provides emergency medical care to a person during a state of emergency.” This would be an important consideration for other countries to follow.

## Recommendations for Dental Implementation in Post-COVID-19 Era

COVID-19 has been an unprecedented experience for mankind. The scale and extent of the pandemic has forced many governments to take drastic and decisive action, resulting in considerable disruption in daily life and damage to global economies. This episode has revealed the need to be better prepared for future pandemics. To this end, educational institutions can take the lead. Dental schools should revise and strengthen education on infection control measures in dental practice. Some areas where universal precaution protocols can be re-evaluated and strengthened include hand hygiene, donning and doffing of PPE, respiratory hygiene/cough etiquette, sterilization of instruments and devices, and disinfection of workplaces. Additionally, with the growing evidence of asymptomatic transmission of COVID-19, infection control practices should be re-examined and improved to prevent cross-infection in a dental setting. Notwithstanding, recent attempts have been made to review the current literature on precautions when providing dental care during the current pandemic, and make recommendations for dental practitioners (24, 25).

The curriculum in dental schools should also be expanded to include competencies in pandemic and disaster relief. Such exposure enable dentists and their dental auxiliaries to augment the existing medical professionals in response to declared medical emergencies. Dentists have already been trained to undertake oral swab procedures and biopsies as part of oral cancer screening. However, in the wake of a pandemic outbreak, dental students should be additionally trained to perform nasopharyngeal and oropharyngeal swabs, as well as saliva sampling procedures. Dentists can also be a part of an effective surveillance network by notifying public health authorities about unusual oral symptoms or clinical presentations detected in questionable frequency in a population. Thus, dentists can facilitate the early detection of a disease outbreak or bioterrorism attack, and prevent mass casualties by prompt interventions.

In addition, voluntary medical work should be made a part of the community dentistry curriculum. In dentistry, clinical outreach programs during undergraduate training are still in their infancy. Such programs should be quickly scaled up. Several studies have reported that overseas voluntary outreach programs are a fulfilling and life-changing experience for dentists (26), and that community-based learning experience brings significant positive outcomes in terms of productivity and higher professional standards of dental students (27). With the expanded level of contact with patients from various strata of society, students will have additional opportunities to see the complexities of social and cultural aspects of their future patients. In addition, students are likely to gain appreciation for alternative career paths in public health as well as volunteer work. These experiences also have a positive impact on their understanding of ethical and social issues related to oral health care (28).

Some dental schools have already included community engagement programs as a part of the dental curriculum. Such engagements instill a sense of voluntarism in the minds of the dental graduates and prepare them to contribute in future disease outbreaks. A study looking at Stony Brook University's humanitarian dental mission to rural Madagascar found that all dental students who participated gained experience and confidence in their clinical ability and increased their speed in performing procedures under demanding conditions (29). Beyond medical training, such programs also nurture team work, communication skills and leadership qualities in young dentists.

Moving forward, it is likely that dental practice will be considerably changed in the post-pandemic era. Therefore, there is a greater need for refresher courses for practicing dentists on infection control in order to adjust with patients' apprehension in the post-COVID-19 world. Dental authorities and dental schools should urgently look into this need and appoint taskforces to develop protocols and appropriate courses for dental practitioners. Concurrently, research on infection control in the dental setting needs to be advanced. In order to formulate best practices, new research should be conducted across all disciplines of dentistry covering all procedures and their respective infection control strategies. The field of public oral health should find new research avenues on community oral health that can provide an insight on the perception and apprehension of patients during hospital visits. Such information would help to revive the financial viability of public hospitals and private clinics. The recommendations for dental implementation in the post-COVID-19 era are summarized in Table 1.

## Occupational Health and Safety

Volunteers are being selfless in providing services amid crisis. However, it should be borne in mind that similar with medical staff, volunteers are also a vulnerable group. Preparation and medical support for volunteers during their placement is a significant aspect of any overseas voluntary work. Lack of understanding and preparation may expose volunteers to certain diseases during community engagement as well as subject them to psychological problems (30). Hence, it is highly advisable that proper training is provided to the volunteers. Volunteers should be trained on proper donning and doffing of PPE. It is important not just to minimize cross-infection but to keep it to zero. Therefore, strict infection control measures such as correct use of gloves and hand hygiene steps should be practiced. Volunteers working in close contact with COVID-19 positive patients should check for proper fit and size of N95 masks. It is very likely for cross-contamination to occur during public mass-scale swabbing operations. A single breach of the chain of infection control will put the whole team in jeopardy leading to quarantine of its members and closure of the medical facility.

Not all volunteers have the same physical and mental strength and the amount of volunteering work that one can engage must be monitored by group leaders and higher level authority. Frequent breaks in between and off days are necessary, in particular for a longer engagement, to support mental and psychosocial well-being of the volunteers. It is also important to provide an understanding of the precautionary safe-distancing measures to be taken with family during and after engagement in risk activities. It is essential that volunteers are mindful of their own and their family's health while serving others.

## Conclusion

Overall, it is commendable that many dentists have risen to the challenge in the fight against COVID-19. The role of the dentist in a pandemic can be beyond dentistry. By virtue of their training and practical experience, dentists can provide services in various ways to reduce the strain on the healthcare sector. Volunteerism in such a time also leaves a positive impact on the individual. Pandemics rarely occur, and practical experience gained will be a lifelong lesson for the volunteer. In fact, the fighting spirit of a volunteer working in risky operations instills a high moral esteem and self-confidence. The rejuvenated personality of the volunteer can prove to be valuable in developing his career in future. The selfless voluntary service will be appreciated by the larger community and future generations. Together we will be able to pull through this crisis and emerge stronger than before.

## Ethics Statement

Written informed consent was obtained by the Ministry of Health Singapore for the publication of any potentially identifiable images or data included in this article.

## Author Contributions

CJS and ML contributed to the conception. CJS, ML, and BTG drafted and critically revised the manuscript. All authors contributed to the article and approved the submitted version.

## Conflict of Interest

The authors declare that the research was conducted in the absence of any commercial or financial relationships that could be construed as a potential conflict of interest.
